# Patterns and Rates of Plastid *rps*12 Gene Evolution Inferred in a Phylogenetic Context using Plastomic Data of Ferns

**DOI:** 10.1038/s41598-020-66219-y

**Published:** 2020-06-10

**Authors:** Shanshan Liu, Zhen Wang, Hui Wang, Yingjuan Su, Ting Wang

**Affiliations:** 1 School of Life Sciences, Sun Yat-sen University, Guangzhou, 510275 China; 2 Fairy Lake Botanical Garden, Shenzhen & Chinese Academy of Sciences, Shenzhen, 518004 China; 3 Research Institute of Sun Yat-sen University in Shenzhen, Shenzhen, 518057 China; 4 College of Life Sciences, South China Agricultural University, Guangzhou, 510642 China

**Keywords:** Molecular evolution, Plant evolution, Plant molecular biology, Evolutionary biology, Sequencing, Genome evolution

## Abstract

The *trans*-splicing *rps*12 gene of fern plastomes (plastid genomes) exhibits a unique structure owing to its variations in intragenic exon location and intron content, and thus, it provides an excellent model system for examining the effect of plastid gene structure on rates and patterns of molecular evolution. In this study, 16 complete fern plastome sequences were newly generated via the Illumina HiSeq sequencing platform. We reconstructed the phylogeny of ferns and inferred the patterns and rates of plastid *rps*12 gene evolution in a phylogenetic context by combining these plastome data with those of previously published fern species. We uncovered the diversity of fern plastome evolution by characterizing the structures of these genomes and obtained a highly supported phylogenetic framework for ferns. Furthermore, our results revealed molecular evolutionary patterns that were completely different from the patterns revealed in previous studies. There were significant differences in the patterns and rates of nucleotide substitutions in both intron-containing and intron-less *rps*12 alleles. Rate heterogeneity between single-copy (SC) and inverted repeat (IR) exons was evident. Unexpectedly, however, IR exons exhibited significantly higher synonymous substitution rates (dS) than SC exons, a pattern that contrasts the regional effect responsible for decreased rates of nucleotide substitutions in IRs. Our results reveal that structural changes in plastid genes have important effects on evolutionary rates, and we propose possible mechanisms to explain the variations in the nucleotide substitution rates of this unusual gene.

## Introduction

Plastid ribosomes are ubiquitous organelles in plant cells and play a vital role in the biosynthesis of proteins. In higher plants, plastid ribosomes contain approximately 60 ribosomal proteins that are encoded in both the plastid and the nuclear genetic compartments^1^. Among the plastid-encoded ribosomal protein gene structures, *rps*12 is the most notable. The plastid ribosomal protein S12 encoded by the *rps*12 gene is a highly conserved protein located in the functional center of the 30S subunit of the ribosome^2^. In fern plastomes (plastid genomes), where *rps*12 is a *trans*-splicin*g* gene, this gene is split into three exons by two introns in most, but not all, ferns, and one intron (intron I) is discontinuous. The first exon of the *rps*12 gene is generally located in the large single-copy (LSC) region, whereas the second and third exons reside in the inverted repeats (IRs); the two IR copies have identical sequences but opposite transcriptional directions. More importantly, the second intron is lacking from the *rps*12 gene of all species belonging to three basal fern lineages: Psilotales, Ophioglossales, and Equisetales^3,4^. Thus, two distinct *rps*12 gene types were identified in ferns based on the presence or absence of intron II, and type I and II genes corresponded to intron-containing and intron-less genes, respectively. Plastid genomic structural alterations, such as inversions, duplications, and gene or intron loss, are often accompanied by an increase in the rate of plastome sequence evolution^5,6^. Therefore, different copy numbers of exons and the presence or absence of introns have garnered substantial interest as an avenue to explore the evolutionary patterns of this unique gene.

Introns are highly stable components of land plant plastomes, and it is widely believed that a basic set of introns was established prior to the divergence between vascular and nonvascular plants, because this intron set is shared by different taxonomic groups (such as charophytes, bryophytes, and spermatophytes)^7^. The intron contents of fern plastomes are also highly conserved, with no gains and few losses during fern evolution^4,8^. The interspecies variation in the intron content of fern plastomes mainly reflects the complete loss of several intron-containing genes in specific lineages (e.g., intron-containing *rps*16 genes are absent in Psilotales, Ophioglossales, and Equisetales^4^), or the presence of intron-less alleles in some lineages (e.g., intron-less *rps*12 genes are present in Psilotales, Ophioglossales, and Equisetales; *Lygodium japonicum* lacks *rpo*C1 introns that are commonly found in other ferns^8^). Intron losses have been associated with elevated substitution rates in plastid genes, as previously described^5,9,10^, but there is an extreme bias in taxon sampling of these studies. The vast majority of these studies are based on angiosperms. Consequently, we cannot determine whether this pattern is a universal phenomenon in the plastome or an independent evolutionary event in certain lineages or genes. The plastid *rps*12 gene in ferns provides an excellent model system to study the effect of intron losses on evolutionary rate and test the generality of this evolutionary pattern.

Another distinctive feature of the *rps*12 gene in ferns is the variation in copy number among its exons. As mentioned previously, in most ferns, the first exon of *rps*12 is located in the LSC, whereas the second and third exons reside in the IRs. It is well known that two completely identical IR regions are prominent structural features of the plastomes of nearly all land plants. The regional effect of the evolutionary pattern of plastid genes, in which the evolutionary rate of genes located in the IRs is lower than that of genes in the single-copy (SC) regions, has been documented with extensive research^11–15^. The sequence identity of IRs can be maintained by copy-dependent DNA repair because when mutations are introduced into one IR copy, the other copy provides a template for error correction^13,16^, thereby suppressing the substitution rate in the IRs. Biased gene conversion, as an efficient mutation-correcting mechanism, can result in a genome with different copy regions that have different mutation rates^16,17^. There are up to 900 genomic copies in a single plastid^18^, whereas the duplicative property of the IRs provides an even greater number of copies; therefore, the frequency of gene conversion in the IRs should be higher than that in the SC regions, and this phenomenon might be responsible for a significantly lower evolutionary rate in the IRs than in the SC regions^14,16^.

However, with a growing quantity of plastome data available for comparative genomics, this hypothesis of the regional effect of the IRs has been refuted by several studies^19–21^. In addition to the impact of IRs on the evolutionary rate, the non-IR localization, locus- and lineage-specific also have significant effects on evolutionary rate heterogeneity in plastomes^19,21–23^. Therefore, we suggest that each of these factors alone cannot sufficiently explain the rates and patterns of molecular evolution in plastid genes. For this reason, we turned our research focus to the split *rps*12 gene because of its location in both the SC and IR regions. The variation in the exon location and intron content of the *rps*12 gene in fern plastomes provides a unique opportunity to explore the effect of gene structure on sequence evolution. To thoroughly understand the pattern of *rps*12 gene evolution in ferns, in the current study, we employed greatly expanded taxon sampling, including 91 fern species, and undertook a broad survey to investigate the impact of structural variation on plastid gene evolution.

## Results and Discussion

### Organization and dynamic structural evolution of plastid genomes in ferns

**Figure 1:**
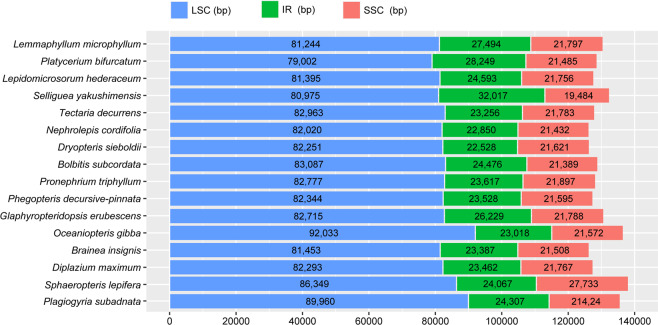
Sizes of each part of 16 fern complete plastome sequences.

Whole-genome sequencing using an Illumina HiSeq platform generated 6,794,240–23,309,670 raw reads for 16 samples. We obtained 6,026,844–21,215,174 clean reads by removing adaptors and low-quality read pairs (Table 1). Following *de novo* and reference-guided assembly strategies, 16 newly sequenced fern plastomes were each assembled into a single circular molecule. Both genome size and GC content were relatively conserved among all species (Table 2). The size of the 16 plastome sequences ranged from 148,928 bp in *Dryopteris sieboldii* to 164,857 bp in *Selliguea yakushimensis*, and all plastomes displayed a typical quadripartite structure consisting of a large-single copy region (LSC, 79,002–92,033 bp), a small single-copy region (SSC, 19,484–27,733 bp), and a pair of inverted repeats (IRs, 22,528–32,017 bp) (Fig. 1 and Table 2). Across all sequenced ferns, there were 84–86 protein coding genes, 27–29 tRNA genes and 4 rRNA genes. The polypods and tree ferns had similar coding gene contents, with a few notable distinctions. Compared with the 84 protein coding genes inferred to be present in the ancestral plastomes of polypods, tree ferns had an additional *psa*M gene that was shared with most other non-polypods. The majority of the sequenced samples displayed typical fern intron contents. The *rps*12 gene in all the sequenced species was classified as type I, and the first exon was located in the LSC, far away from the second and third exons of *rps*12, which were present in two copies in the IRs.

**Table 1:** Summary of the sequencing data for 16 fern species.

Species	Raw data (G)	Clean data (G)	Raw Reads	Clean reads	Coverage (×)	Accession number
*L. microphyllum*	2.43	2.08	8,083,733	6,927,768	198.45	MN623356
*P. bifurcatum*	6.99	6.36	23,309,670	21,215,174	552.65	MN623367
*L. hederaceum*	2.28	2.21	7,585,298	7,066,889	260.67	MN623364
*S. yakushimensis*	2.94	2.71	9,815,752	9,033,527	284.70	MN623352
*T. decurrens*	2.17	2.05	7,228,697	6,826,579	24.29	MN623363
*N. cordifolia*	2.04	1.81	6,794,240	6,026,844	85.16	MN623365
*D. sieboldii*	3.26	3.00	10,851,235	10,014,316	221.45	MN623354
*B. subcordata*	2.48	2.28	8,276,022	7,604,656	127.23	MN623358
*P. triphyllum*	5.79	5.37	19,289,987	17,902,643	356.00	MN623361
*P. decursive-pinnata*	3.22	2.86	10,736,184	9,531,427	79.56	MN623353
*G. erubescens*	2.89	2.63	9,643,034	8,770,301	1196.58	MN623355
*O. gibba*	3.25	3.06	11,775,566	11,370,848	125.57	MN623360
*B. insignis*	3.86	3.58	12,880,011	11,926,722	178.90	MN623366
*D. maximum*	3.13	2.87	10,430,597	9,565,536	128.62	MN623359
*S. lepifera*	3.10	2.87	10,346,974	9,561,719	253.00	MN623357
*P. subadnata*	2.80	2.56	9,342,117	8,540,938	393.80	MN623362

**Table 2:** Plastome features of the sequenced species. Plus and minus signs denote genes that are present and absent, respectively, in the corresponding species. ψ represents pseudogenes.

Species	Genome size (bp)	LSC (bp)	IR (bp)	SSC (bp)	GC %	Gene	*trn*R-UCG	*trn*V-UAC	*trn*T-UGU	*trn*N-GUU
*L. microphyllum*	158,029	81,244	27,494	21,797	41.83	132	+	+	+	+
*P. bifurcatum*	156,985	79,002	28,249	21,485	39.91	130	−	−	+	+
*L. hederaceum*	152,337	81,395	24,593	21,756	42.65	132	+	+	+	+
*S. yakushimensis*	164,857	80,975	32,017	19,848	40.80	135	+	+	+	+
*T. decurrens*	151,258	82,963	23,256	21,783	41.98	129	−	+	+	−
*N. cordifolia*	149,152	82,020	22,850	21,432	39.36	131	−	+	+	+
*D. sieboldii*	148,928	82,251	22,528	21,621	43.08	131	+	−	+	+
*B. subcordata*	153,428	83,087	24,476	21,389	42.39	132	+	+	+	+
*P. triphyllum*	151,908	82,777	23,617	21,897	42.82	132	+	+	+	+
*P. decursive-pinnata*	150,995	82,344	23,530	21,591	42.37	132	+	+	+	+
*G. erubescens*	156,961	82,715	26,229	21,788	43.15	132	+	+	+	+
*O. gibba*	159,641	92,033	23,018	21,572	43.53	132	+	+	+	+
*B. insignis*	149,734	81,453	23,387	21,508	41.40	131	+	−	+	+
*D. maximum*	150,984	82,293	23,462	21,767	43.90	132	+	+	+	+
*S. lepifera*	162,216	86,349	24,067	27,733	40.80	132	+	+	ψ	+
*P. subadnata*	159,998	89,960	24,307	21,424	42.94	132	+	+	ψ	+

Although the plastome structure remained relatively stable, some species showed exceptional variability in tRNA gene content (Table 2). Several tRNA gene losses from multiple independent lineages were detected, including the loss of *trn*R-UCG in *Platycerium bifurcatum*, *Tectaria decurrens*, and *Nephrolepis cordifolia*; the loss of *trn*V-UAC in *P. bifurcatum*, *D. sieboldii*, and *Brainea insignis*; the loss of *trn*N-GUU in *T. decurrens*; and the pseudogenization of *trn*T-UGU in *Sphaeropteris lepifera* and *Plagiogyria subadnata* (Table 2). All of these genes have also been parallelly lost in other polypods plastomes with the exception of the *trn*N. The *trn*N is one of the core set genes contained in IRs, generally adjacent to either *ndh*F or *chl*L at the IR/SSC borders. In ferns, IRs are generally considered to be the most stable part of the plastome because genomic rearrangement rarely occurs in these regions. However, in contrast, a recent study indicated that the IR sequences and gene contents were highly variable in polypods^24^. Our results are in accordance with the latter findings and provide additional evidence for the dynamic evolution of IR regions among closely related polypod plastomes.

**Figure 2:**
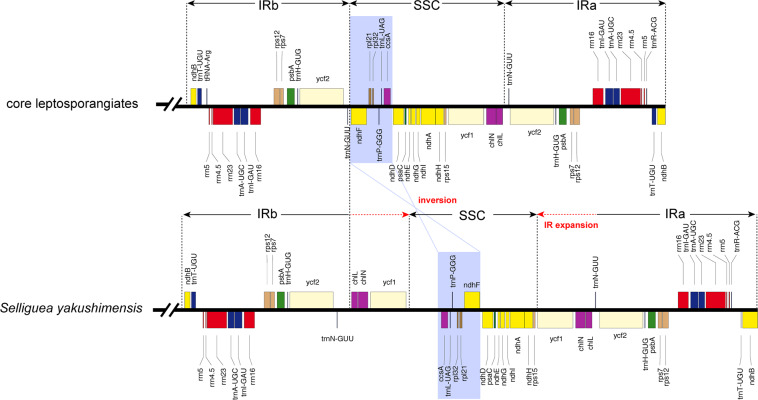
Unique structural changes in the plastome of *Selliguea yakushimensis* compared to those of other core leptosporangiates. Genes are represented by colored boxes above or below of the black chromosome bar according to the direction of transcription. The novel inversion that occurs in the *S. yakushimensis* is shown in a purple box. The red dashed arrow indicates the range of the IR expansion in the *S. yakushimensis* plastome.

Polypods are the lineage of most derived ferns that diversified in the Cretaceous period, displaying an ecologically opportunistic response to the diversification of angiosperms^25^. The plastomes of polypods have undergone multiple complex genomic reconfigurations during fern evolution, and thus, their plastomes differ substantially from the plastomes of basal ferns (Psilotales, Ophioglossales, Marattiales, and Equisetales). Plastome evolution among polypods is considered relatively static compared with that in lineages other than polypods^26^. Surprisingly, distinct genome organizations were identified in *S. yakushimensis* based on inversions and IR boundary variation. A major variation in the *S. yakushimensis* plastome relative to the genomes of other core leptosporangiates is the altered location from the *ndh*F-ccsA (Fig. 2). In addition, IR expansion into the SSC resulted in a duplication of the *ycf*1, *chl*L,  and *chl*N genes in the *S. yakushimensis* plastome. The extent of IR expansion in the *S. yakushimensis* plastomes is unprecedented among ferns. The IRs in *S. yakushimensis* are up to 32 kb in length (Fig. 1 and Table 2), whereas the longest IR found previously was 29 kb in *Cibotium barometz*^27^. These rare genome structure variations in polypods were detected for the first time.

### Phylogenetic analyses

**Figure 3:**
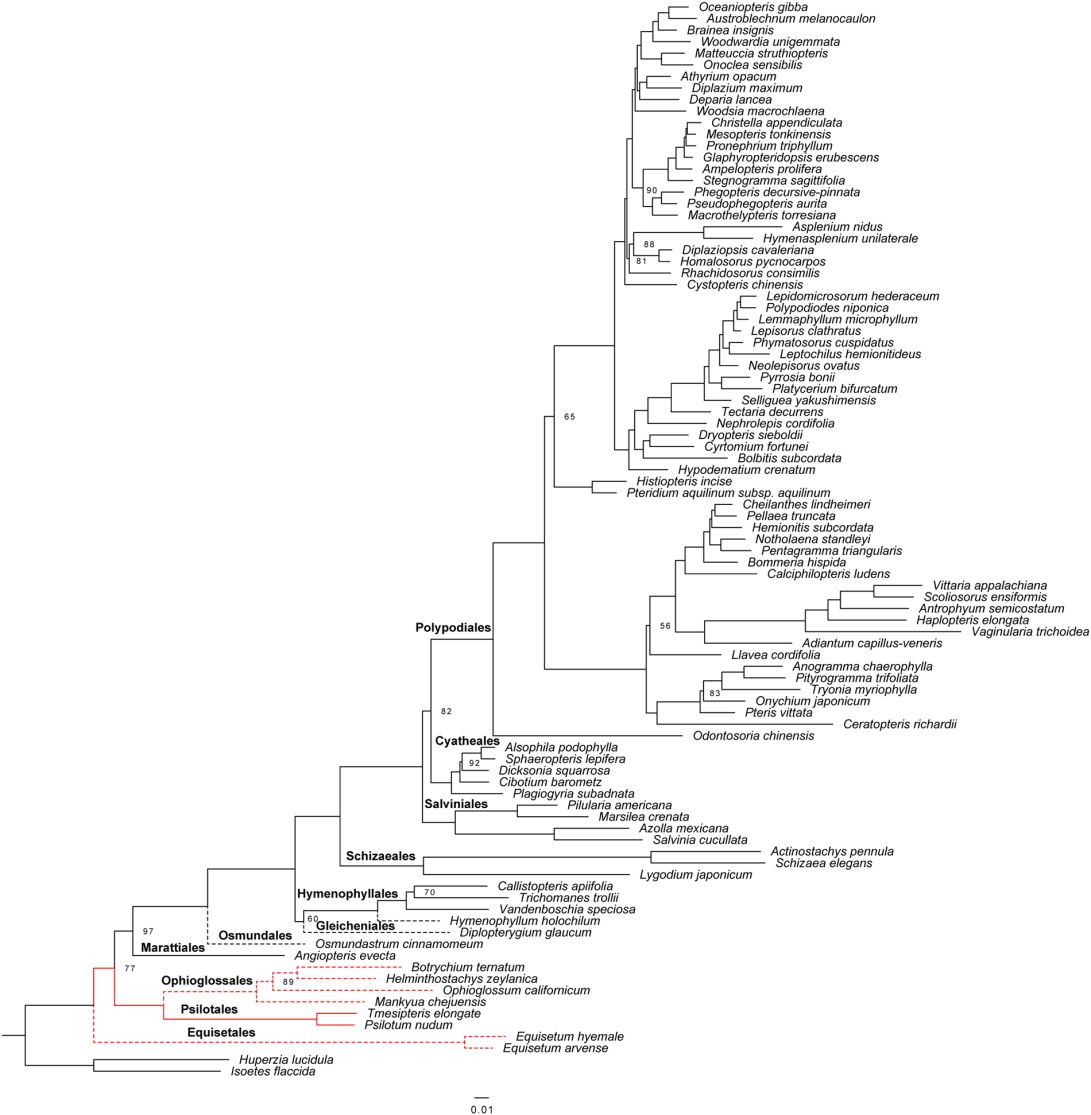
Phylogram showing intron losses and the distribution of the *rps*12 gene in fern plastomes. The topology was based on an ML tree generated from 50 concatenated protein-coding genes from 91 fern and two outgroup plastomes. Only nodes with bootstrap support values less than 100% are shown. The absence of the *rps*12 intron is indicated with a red line; the dashed line denotes that all the exons of *rps*12 were present in only one copy.

Both the 93- and 84-taxon datasets based on 50 protein-coding genes showed consistent phylogenetic framework, differing only in the support values for some nodes (Fig. 3 and Supplementary Fig. S1). Along the backbone, Equisetales was uncovered as a sister clade to the remaining ferns with strong support, followed by a highly supported joint Ophioglossales + Psilotales clade, itself sister to a clade with Marattiales (Fig. 3). The phylogenetic relationships among these four basal fern orders are the most debated topics in fern phylogeny. Most previous studies using nuclear genes^28–30^, a combination of mitochondrial and plastid sequences^31^, several plastid genes^32,33^, and whole plastome sequences also obtained this topology^34,35^. In contrast, other phyloplastomic studies tend to support grouping Equisetales and Ophioglossales + Psilotales together as a monophyletic group and sister to the remaining ferns^36,37^. In addition, the recent phylotranscriptomic analyses have also revealed a distinct topology of relationships among the basal fern orders, showing that Equisetales is the sister group to all other ferns, whereas Marattiales and Psilotales + Ophioglossales form a monophyletic group^38^. Given that plastid genes generally evolve more slowly than the nuclear genes, these topological differences may be due to different numbers of phylogenetically informative sites contained within the diverse molecular data^39^.

The diversification of leptosporangiates occurred after that of eusporangiate ferns, within which Osmundales was the earliest-diverging lineage, and then, Gleicheniales and Hymenophyllales formed a monophyletic group sister to the remaining non-Osmundales ferns (Fig. 3). The phylogenetic position of Hymenophyllales remains debated. Most phylogenetic analyses have depicted two alternative relationships: Hymenophyllales as a sister clade to Gleicheniales + the rest of non-Osmundales leptosporangiates^25,32,33,40–43^, or Hymenophyllales and Gleicheniales together form a clade that is sister to the remaining non-Osmundales leptosporangiates^29,38,44^. Interestingly, both topologies have been found in more recent plastid phylogeny reconstructions using different data types and partition schemes^37^. Kuo *et al*.^37^ showed that the use of plastome organization features also fails to provide additional support for either of these two topologies. This phylogenetic uncertainty is probably due to sparse taxon sampling, data types, model selection, and tree inference methods. Although the relative positions of the order Hymenophyllales remains inconclusive, our results should not be ignored based on the feasibility and effectiveness of plastomes for inferring phylogenies.

As in previous studies, Schizaeales was clearly identified as the sister clade to the core leptosporangiates^29,33,36,38,44^. In the core leptosporangiates, Salviniales and Cyatheales are successive sisters to Polypodiales, and each of these orders is clearly monophyletic, with moderate to high support (Fig. 3 and Supplementary Fig. S1). Our results showed that the earliest-diverging clade of Polypodiales is Lindsaeaceae, followed by Pteridaceae. These clades are well supported as monophyletic. Although Dennstaedtiaceae was weakly supported as the sister lineage to the eupolypods in our study, the relationship found here is congruent with the findings of most recent studies^28,36,38,45^ (Fig. 3 and Supplementary Fig. S1). Eupolypods account for well over half of the extant fern diversity, and determining their sister group has been difficult. Earlier studies reported that Pteridaceae, instead of Dennstaedtiaceae, was sister to the eupolypods; however, the relationships among these three lineages were not well-resolved because of low support^41,42^. These relationships need to be further studied to ascertain which family is the sister lineage to the eupolypods.

### Impact of intron loss on the *rps*12 evolutionary rate

The occurrence of *rps*12 intron loss in three basal fern lineages is considered to be an important evolutionary event in ferns. If the phylogenetic relationships among ferns are indeed consistent with our analysis, which recovered Equisetales as a sister clade to the remaining ferns, then the intron of the *rps*12 gene would have been independently lost at least twice during fern evolution^4^. Research suggests that the rate of plastome sequence evolution is generally affected by structural changes^5,6^; thus, we sought to investigate the impact of intron loss on the evolutionary rate of the *rps*12 genes.

**Figure 4:**
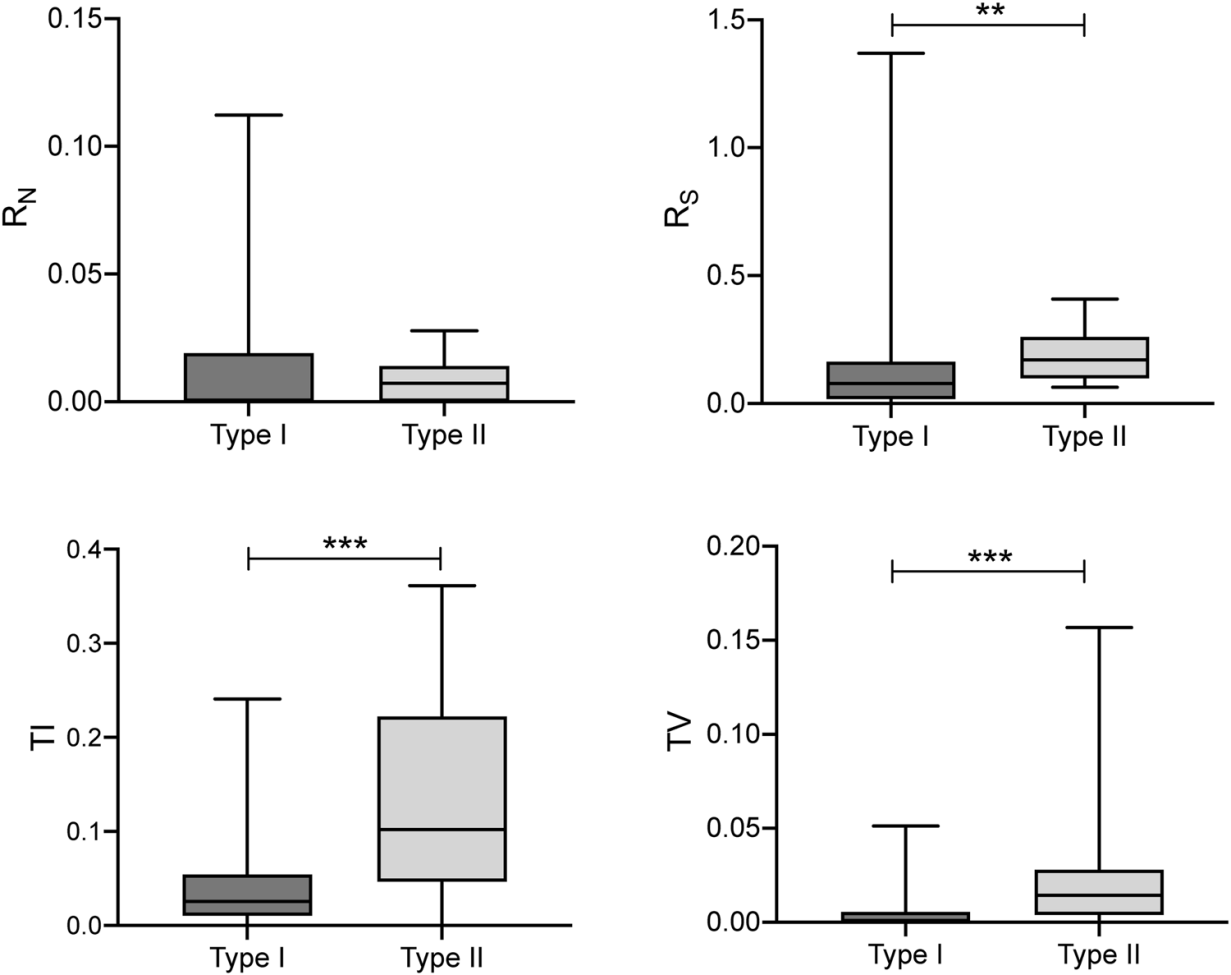
Comparison of R_N_, R_S_, TI, and TV rates between intron-containing (type I) and intron-less (type II) *rps*12 genes in ferns. Asterisks indicate ^(**)^*P* < 0.01 and ^(***)^*P* < 0.001.

Branch length in a phylogenetic tree represents an estimate of the amount of sequence divergence in the corresponding lineage, which is equal to the product of the absolute substitution rate and time^46^. For this reason, we cannot ignore the timescale over which molecular rates change in comparisons of substitution rates among taxa. Therefore, we tested for an intron effect on substitution rate changes by comparing the absolute substitution rates of the type I and type II genes. Our results showed that the *trans*-splicing *rps*12 genes in all examined species were highly conserved with a size of 372 bp, encoding a total of 123 amino acids. The differences in the rates between the two types of genes were mainly reflected in the rates of synonymous substitution (R_S_). Wilcoxon rank sum tests showed that the values of R_S_ for type II genes were significantly higher than those for type I genes (*P* < 0.01), whereas the values of the rates of nonsynonymous substitution (R_N_) were not significantly different (*P* = 0.8019) (Fig. 4; Supplementary Fig. S2 and Table S1). Moreover, in order to detect whether the selection pressures acting on the two types of genes were significantly different, we compared dN/dS ratios in a phylogenic context via a model-based approach. A likelihood ratio test (LRT) was used to compare the fits of two models: the null model, where values of dN/dS were not significantly different between type I and type II, and the alternative model, where type I had different dN/dS ratios relative to type II. Overall, the alternative model was significantly different from the null model (*P* < 0.05).

Currently, the most widely accepted mechanism of intron loss is a reverse transcriptase (RT)-mediated model (namely, retroprocessing), which is a process of integrating intron-less cDNA generated by reverse transcription of the corresponding mRNA into the genome by homologous recombination^47–49^. Another two possible mechanisms of intron loss include genomic deletion^50^ and exonization of intronic sequences^51^. In these cases, the intron is often removed imprecisely, changing the intron/exon borders. Consequently, the *rps*12 intron loss that occurred in the plastomes of ferns is indicative of the first mechanism because the exon boundaries of the intron-less genes have been shown to be perfectly matched to that of the intron-containing genes. Previous studies have reported that the accelerated evolution of *clp*P1 in many seed plant lineages is associated with intron loss^9,10,52^. A possible explanation for this acceleration in *clp*P1 evolution is that reverse transcriptases and/or RNA polymerases have higher error rates during retroprocessing^53^. Although the rapid evolution of the *clp*P1 gene could also relate to a hybrid effect (e.g., duplications, indels, and pseudogenization), we found a significantly higher R_S_ for type II genes than for type I genes in the fairly conserved gene *rps*12, indicating that accelerated gene evolutionary rates are correlated with loss of introns. Therefore, this phenomenon may be a prevalent genome-wide pattern.

Furthermore, we analyzed the two well-known types of genetic mutations, transitions (TIs) and transversions (TVs), to test whether there were differences in mutation pattern between type I and type II genes. Our results showed that TIs occurred more frequently than TVs in both type I and type II genes (Fig. 4; Supplementary Fig. S2 and Table S2). From the perspective of natural selection, this “transition bias” phenomenon is considered to indicate that selection disfavors transversions, as transversions are more likely to alter the amino acid sequence of proteins than transitions^54^. Comparison between the TI and TV values showed that both values were significantly higher in type II than in type I genes (*P* < 0.001) (Fig. 4; Supplementary Fig. S2 and Table S2). An LRT was also implemented to identify rate shifts between the type I and type II branches, and the results indicated that TI/TV was significantly different in the type I branches than in the type II branches (*P* < 0.001). That is, transition and transversion events that occurred in type II genes resulted in more synonymous changes than those in type I genes. This finding may explain the significant change in R_S_ between the type I and type II genes, whereas R_N_ did not exhibit significant changes.

### Complicated rate variation in *rps*12 exons

**Figure 5:**
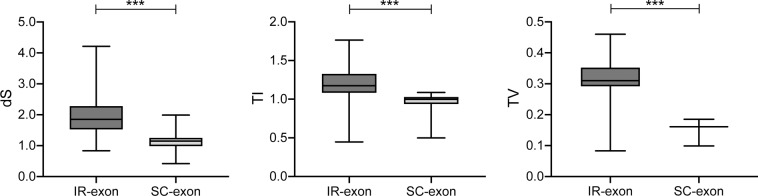
Comparison of dS, TI, and TV values between IR and SC exons of *rps*12 genes in ferns. Asterisks indicate ^(***)^*P* < 0.001.

Due to a series of genomic inversions across the fern phylogeny, 84 of 93 sampled species showed the same pattern of exon distribution, which was divided into IR and SC exons. The consensus sequences of the SC and IR exons of the fern *rps*12 genes were 114 bp (encoding the 1st to 38th amino acids) and 232 bp in length (encoding the 39th to 123rd amino acids), respectively. Since nonsynonymous mutations are more strongly affected by selective constraints, while synonymous mutations are largely invisible to natural selection, a comparison of the synonymous substitution rates provides a better understanding of DNA sequence evolution^55^. In order to compare with previous research more intuitively, we used the same pairwise comparison method as the previous studies when investigating the difference between the rates of IR and SC exons^12,21^. Our results revealed a more complicated rate variation in the *rps*12 exons than expected. The dS values for the IR exon were significantly higher than those for the SC exon (*P* < 0.001), a pattern that contrasts the regional effect responsible for decreased rates of nucleotide substitutions in the IRs (Fig. 5; Supplementary Table S3). The results of an LRT between the one-rate and two-rate models showed significant rate changes relative to the position of an exon in either the IR or SC region (*P* < 0.01).

A previous study indicated that the total substitution rates decreased after gene translocation into the IRs by using a model-based approach across the fern phylogeny^11^. Moreover, Zhu *et al*.^12^ compared the dS values of IR and SC genes across land plants using the pairwise estimation method, including some representative fern species. Based on very limited taxon sampling, they observed that former IR genes that moved into the SC region due to genome rearrangements underwent corresponding dS accelerations. However, we discovered that the IR exons of a given gene in fern species had, in fact, significantly higher dS rates on average than the corresponding SC exons. The most substantial distinction between our study and previous studies is that we compared the IR exons of *rps*12 with the SC exons of the same gene rather than other SC genes. Furthermore, we employed vastly expanded taxon sampling that included not only the major fern lineages but also additional species of derived lineages. Comparing the rate changes in exons located in different regions of a given gene can effectively avoid rate heterogeneity caused by lineage effects^8^. The intragenic location changes in the *rps*12 exons present a unique opportunity to test the effect of IRs on evolutionary rate variation. Thus, the pattern of substitution rate inhibition in the IRs may not be applicable to the fern *rps*12 gene. Indeed, several studies have shown that a pattern of decreased IR substitution rates is not universally suitable among vascular plants, as increased IR substitution rates were previously observed in some genes of *Pelargonium*^19,21^, *Plantago*, and *Silene*^12^. Several possible mechanisms have previously been proposed to explain substitution rate increases in plastid genes. These abnormally evolving genes could result from local hypermutation potentially induced by a high level of error-prone double-strand break repair^12^. A subsequent study by Weng *et al*.^21^ expanded taxon sampling to characterize rates and patterns of evolution in *Pelargonium*, and the results showed that the anomalous rate acceleration observed in *Pelargonium* plastomes could be explained by a mixture of locus-specific, lineage-specific, and IR-dependent effects.

Thus, the mechanisms responsible for generating substitution rate variation may be different in each case. Considering that homologous recombination could enhance copy-correction activity in the IRs via gene conversion, the most direct explanation is the reduction in recombinant activity in IRs. Furthermore, a model of recombination repair^16,56^ could serve as another possible explanation for their observations of increased dS. Recombination repair is also known as post-replication repair^56^, which is a process of repairing impaired molecules by using undamaged molecules as donors, and if this repair is error-prone, single base pair substitutions will be generated because the repair process involves gap-filling DNA synthesis^16^. Additional copies of IR exons in a cell or organelle can act as donors to ensure the efficiency of recombination repair. Consequently, we speculate that recombinant activity and recombination repair may have larger effects than the IR on the substitution rate variation in the *rps*12 gene.

Furthermore, values of TI and TV for SC and IR exons were also estimated. The results showed that both the TI and TV values in the SC exons were significantly higher than those in the IR exons (Fig. 5; Supplementary Table S3), and an LRT rejected the one-rate model in favor of the two-rate model (*P* < 0.001). Likewise, comparisons of the DNA sequences of the *rps*12 gene showed that the base composition in the two exons was not uniform and an excess of transitional over transversional substitutions was present. One reason for the transition bias observed in these exons could be a mutational bias due to the intrinsic properties of DNA, as purines and pyrimidines have different conformational sizes.

## Materials and Methods

### Taxon sampling and DNA extraction

Ferns are a species-rich lineage of vascular plants, occupying a high diversity of ecological niches, and are a major component of the earth’s land flora^57^. However, only 132 complete plastomes are available from GenBank. All 132 complete fern plastome sequences in the NCBI RefSeq collection as of 10 Feb 2019 were downloaded from GenBank, and the annotated coding sequences and the number of exons in *rps*12 were extracted from these sequences. Because the *rps*12 gene is highly conserved in fern plastomes and no substantial variation in the *rps*12 sequence was observed among congeners, only one sample was chosen from each genus (except for two samples that were selected from *Equisetum*) among the previously published fern plastomes to reduce redundancy in the dataset. Then, additional plastome sequences from 16 fern species were sequenced to ensure the coverage of additional fern clades. The increased sampling was undertaken to better understand the evolutionary patterns of *rps*12 gene in ferns. Sequenced species mainly included tree ferns (Cyatheales) and polypod ferns (Polypodiales), which contain most of the extant fern diversity. This sampling strategy resulted in 93 samples representing all 11 extant fern orders and 32 families and two plastomes of lycophytes (outgroup) (Supplementary Table S4).

In this study, fresh leaves of 16 newly sequenced fern species were sampled from Wuhan Botanical Garden, Chinese Academy of Sciences (CSA), and Fairy Lake Botanical Garden (CSA), respectively (Table 3). Samples were taken from young leaves of each plant and were either flash-frozen in liquid nitrogen or placed in paper envelopes and dried with silica gel. Genomic DNA was extracted from the silica-dried or lyophilized leaf tissue of each sample using the Plant Genomic DNA Kit (Tiangen Biotech., Beijing, China). The DNA concentration and purity assessments were performed using agarose gel electrophoresis and a NanoDrop spectrophotometer (Thermo Scientific, Carlsbad, CA, USA). Isolations with concentrations  ≥ 150 ng/μl were chosen for Illumina sequencing.

**Table 3:** Taxa sampled in this study. FLBG, Fairy Lake Botanical Garden, Chinese Academy of Sciences (CSA); WBG, Wuhan Botanical Garden, CAS.

Family	Genus	Species	Sampling site
Polypodiaceae	*Lemmaphyllum*	*L. microphyllum*	FLBG
	*Platycerium*	*P. bifurcatum*	FLBG
	*Lepidomicrosorum*	*L. hederaceum*	WBG
	*Selliguea*	*S. yakushimensis*	WBG
Thelypteridaceae	*Pronephrium*	*P. triphyllum*	FLBG
	*Phegopteris*	*P. decursive-pinnata*	FLBG
	*Glaphyropteridopsis*	*G. erubescens*	WBG
Dryopteridaceae	*Bolbitis*	*B. subcordata*	FLBG
	*Dryopteris*	*D. sieboldii*	FLBG
Blechnaceae	*Oceaniopteris*	*O. gibba*	FLBG
	*Brainea*	*B. insignis*	FLBG
Tectariaceae	*Tectaria*	*T. decurrens*	FLBG
Nephrolepidaceae	*Nephrolepis*	*N. cordifolia*	FLBG
Athyriaceae	*Diplazium*	*D. maximum*	FLBG
Cyatheaceae	*Sphaeropteris*	*S. lepifera*	FLBG
Plagiogyriaceae	*Plagiogyria*	*P. subadnata*	WBG

### Genome sequencing, assembly, and annotation

Genomic DNA was sheared using a Covaris M220 focused-ultrasonication device (Covaris Inc., MS, USA) to a mean fragment size of 300 bp. Paired-end libraries were prepared using the NEBNext Ultra DNA Library Prep Kit (New England Biolabs, Ipswich, MA) for sequencing on an Illumina HiSeq 4000 platform (Illumina Inc., San Diego, CA, USA). Subsequently, 150 bp paired-end reads were produced with an insert size of ~300 bp. Following enrichment, we obtained the raw data for 16 species, ranging from 2.04–6.99 Gb (Table 1). Raw data were then filtered, and adaptor sequences were removed with Trimmomatic v0.33^58^. Trimming was performed from both ends of each read, removing all bases with a quality lower than Q20, and keeping only reads of 50 nt or longer. The quality-filtered reads were subjected to *de novo* assembly using Velvet v1.2.08^59^. Each assembled contig was BLAST searched against previously reported fern plastomes to identify plastid contigs. Gaps that remained in the assembled draft sequence were filled by polymerase chain reaction (PCR) using specific primers that were designed from the regions flanking the gaps.

We annotated plastomes in DOGMA^60^. Plastid genes were corrected in each draft genome by performing homology searches with BLASTX or BLASTN against the previously published fern plastomes. Genes were annotated as pseudogenes if they showed disruptions in reading frames or frameshifts caused by nontriplet insertions or deletions. Transfer RNA (tRNA) genes were verified using two online programs, ARAGORN^61^ and tRNAscan-SE^62^. Plastome maps for all species were drawn using OGDRAW^63^. All fully annotated plastome sequences were deposited in NCBI GenBank under accession numbers MN623352–MN623367 (Table 1).

### Phylogenetic analyses

Phylogenetic analyses were performed on two separate protein-coding gene matrices. The first included 50 shared genes from all the sampled taxa. The second dataset included 50 gene sequences from 84 taxa. This matrix included the same genes as the first dataset except that those taxa with a single copy of *rps*12 were removed (Supplementary Table S4). All sequence alignments for each gene were conducted using MAFFT v7.310^64^, and trimmed to exclude poorly aligned positions using Gblocks v0.91b^65^ with default settings. The trimmed alignments were concatenated in SequenceMatrix v1.0^66^. Phylogenetic trees from the concatenated dataset were estimated using the maximum likelihood (ML) method in RAxML v8.2.4^67^ with the standard GTRGAMMA model by 1,000 rapid bootstrap replicates.

### Estimation of evolutionary rates

Nucleotide sequences for each part exon of the *rps*12 gene from 91 fern species and two lycophyte outgroups, *Huperzia lucidula* and *Isoetes flaccida*, were extracted from plastomes and spliced into a complete coding region. For this step, the exon/intron boundaries of each sample must be carefully examined, and incorrectly annotated genes were manually adjusted if necessary because misalignment would result in the erroneous inference of substitutions rates. The coding sequences of the *rps*12 gene were aligned at the protein level using the Align Codon option of ClustalW as implemented in MEGA7^68^.

Absolute substitution rates for *rps*12 were calculated according to the methods that have been described previously^69,70^. In brief, divergence times for all nodes within ferns were estimated using the penalized likelihood approach in the program r8s^71^, with a time constraint of 354 million years for the crown group age of ferns^25^. The ML tree constructed from the dataset of all sampled taxa was used as the starting tree. We performed 100 bootstrap replicates to calculate standard errors for the divergence time of each node. The branch lengths of dS (number of substitutions per synonymous site) and dN (number of substitutions per nonsynonymous site) trees were computed using the codeml module in PAML v4.8^72^. The standard errors of dN and dS branches were calculated from the standard errors of their corresponding total branch lengths as reported by PAML. Absolute rates of synonymous (R_S_) and nonsynonymous (R_N_) substitution for each branch were calculated by dividing the synonymous/nonsynonymous branch length by the length of time corresponding to that branch.

To compare the nucleotide mutation patterns of the two gene types, the TI and TV rates for *rps*12 were also estimated in the phylogenetic context of ferns using the HKY85 model (the local parameters option) as implemented in HyPhy^73^. The significance of the differences in R_S_, R_N_, TI, and TV between the type I and type II branches were evaluated using Wilcoxon rank sum tests. We used a model-based method to test whether the dN/dS changes differed between the type I and type II branches in a phylogenetic context. The phylogenetic tree of all 93 species generated using ML was used as a constraint tree. The null model, where one dN/dS ratio was fixed across ferns, was compared with an alternative model, where the type I and type II clades were allowed to have different dN/dS ratios. LRTs were performed in PAML v4.8^72^ to compare the goodness of fit of the two models. We also tested for possible rate heterogeneity in the TI/TV ratios across different branches in a phylogenetic context. The LRT scheme for detecting a significant difference in TI/TV between type I and type II genes was conducted using the HKY85 model in HyPhy^73^, in which the null and alternative models specified were the same as those used when detecting dN/dS.

Furthermore, to investigate whether the evolutionary rate variations in the *rps*12 exon corresponded to its location, that is, the IR or SC regions, we performed a parallel analysis of 84 species to determine the effects of IR location on the substitution rate of a given gene. Nine samples were excluded from this analysis because their *rps*12 exon sequences were all single copies (Supplementary Table S4). Based on their locations, the *rps*12 exon sequences were extracted from the plastomes and aligned at the protein level, and the alignments were concatenated separately. The pairwise dS rates for the IR and SC exons between the two lycophytes and each fern species were calculated using the codeml module in PAML v4.8^72^ (seqtype = 1, runmode = −2, and CodonFreq = 2 in the codeml.ctl files). The ML tree inferred from 50 plastid genes of 82 taxa was used as a constraint tree. Likewise, we calculated the pairwise TI and TV of the two exons by using the HKY85 model implemented in HyPhy to compare the nucleotide mutation patterns. Wilcoxon rank sum tests were used to test the rate differences between the SC and IR exons. To investigate whether the dN/dS changes in exons corresponded to their location, on the phylogeny, a model-based rate analysis was used. LRTs were performed using the MG94xHKY85 codon model in HyPhy^73^, in which the one-rate model specified a shared dN/dS across the whole gene, whereas the two-rate model allowed the IR and SC exons to have different dN/dS values. The same setting was applied to the LRTs for TI/TV, except that a HKY85 substitution model was applied.

## Supplementary information

Supplementary Figures.

Supplementary Tables.

## Data Availability

The complete plastome sequences generated in current study are available in GenBank, https://www.ncbi.nlm.nih.gov/genbank/ (accession numbers are described in the text).
