# Supplementary material for: Patterns and Rates of Plastid rps12 Gene Evolution Inferred in a Phylogenetic Context using Plastomic Data of Ferns
Source: Sci Rep. 2020 Jun 10;10:9394. doi: 10.1038/s41598-020-66219-y (PMC7287138; doi:10.1038/s41598-020-66219-y)
Supplement: Supplementary file 1 — Supplementary Figures. [file 41598_2020_66219_MOESM1_ESM.docx]

**Patterns and Rates of plastid *rps*12 Gene Evolution Inferred in a Phylogenetic Context using Plastomic Data of Ferns**

**Shanshan Liu**^1^**,** **Zhen Wang**^1^**,** **Hui Wang**^2^**,** **Yingjuan Su**^1,^^3,^* **& Ting Wang**^4,^*

^1^School of Life Sciences, Sun Yat-sen University, Guangzhou, 510275, China

^2^Fairy Lake Botanical Garden, Shenzhen & Chinese Academy of Sciences, Shenzhen, 518004, China

^3^Research Institute of Sun Yat-sen University in Shenzhen, Shenzhen, 518057, China

^4^College of Life Sciences, South China Agricultural University, Guangzhou, 510642, China

*****Corresponding author: suyj@mail.sysu.edu.cn; tingwang@scau.edu.cn


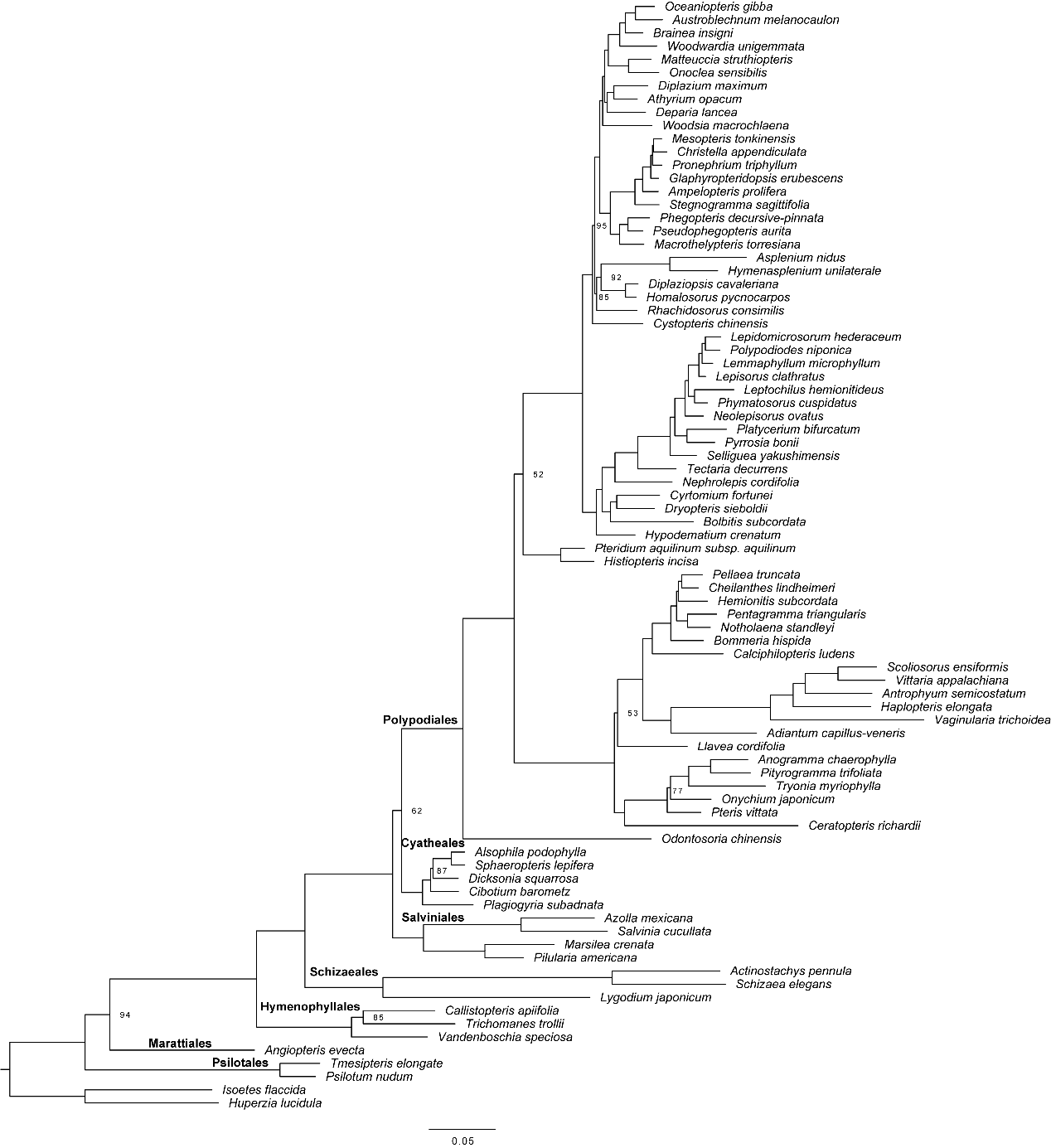


**Figure S1.** Phylogenetic relationships among 82 selected taxa of ferns used as a constraint tree for analyses of rate variation of in the *rps*12 exon. Only nodes with bootstrap support values less than 100% are shown.


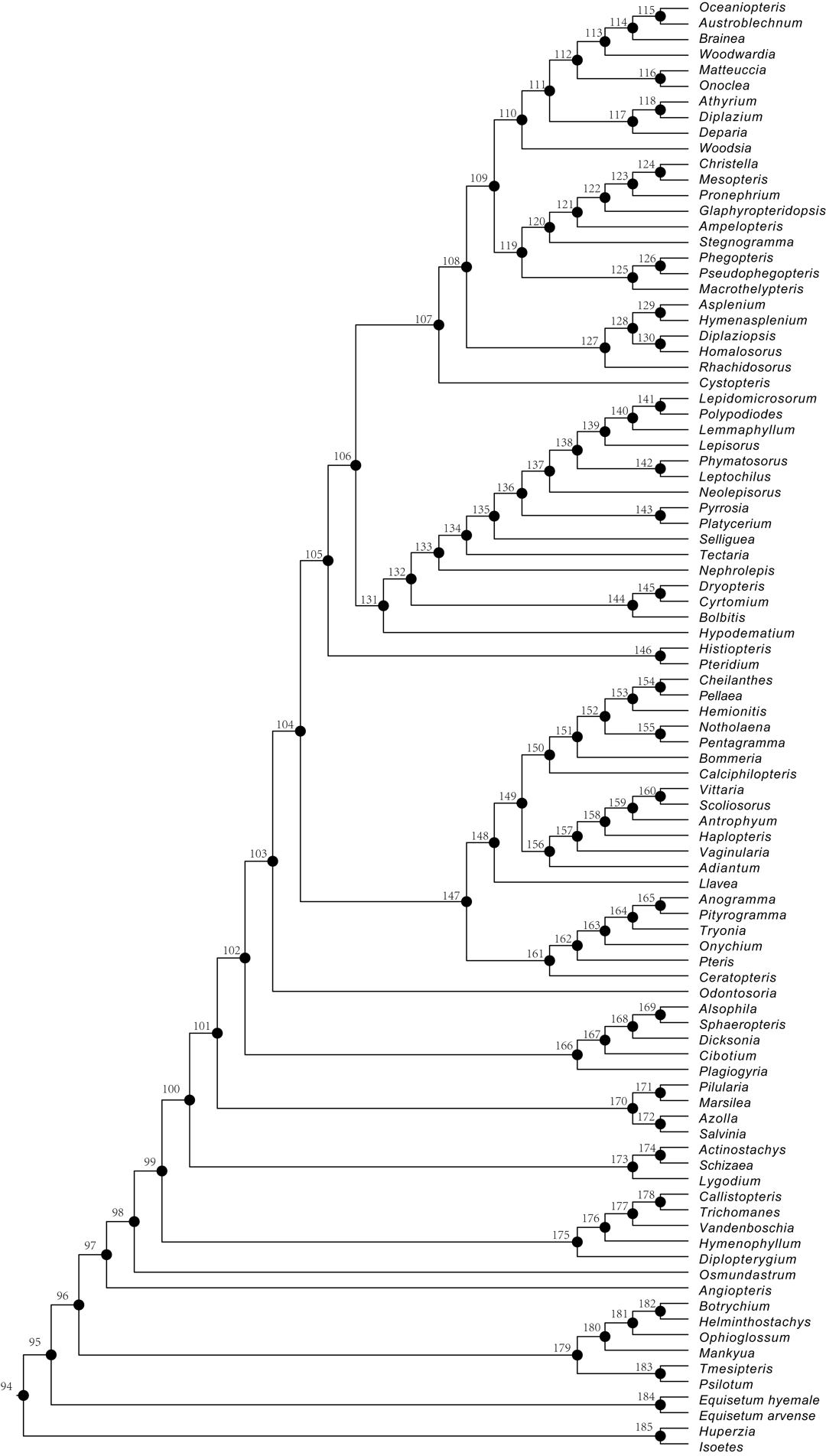


**Figure S2**. Node information used for the estimation of absolute rates for each branch of the phylogenetic tree. Internal nodes are labeled 95–185.
